# Plasma neurofilament light chain levels in chemotherapy‐induced peripheral neurotoxicity according to type of anticancer drug

**DOI:** 10.1111/ene.16369

**Published:** 2024-07-01

**Authors:** Roser Velasco, Carla Marco, Eva Domingo‐Domenech, Agostina Stradella, Cristina Santos, Berta Laquente, German Ferrer, Andreas A. Argyriou, Jordi Bruna

**Affiliations:** ^1^ Neuro‐Oncology Unit of Institut d´Investigació Biomèdica de Bellvitge, Department of Neurology Hospital Universitari de Bellvitge–Institut Català d'Oncologia Barcelona Spain; ^2^ Department of Cell Biology, Physiology, and Immunology Institute of Neurosciences, Universitat Autònoma de Barcelona Bellaterra Spain; ^3^ Department of Haemathology, Catalan Institute of Oncology L'Hospitalet de Llobregat, Institut d´Investigació Biomèdica de Bellvitge Barcelona Spain; ^4^ Department of Medical Oncology, Catalan Institute of Oncology L'Hospitalet de Llobregat, Institut d´Investigació Biomèdica de Bellvitge Barcelona Spain; ^5^ Neurology Department Agios Andreas General Hospital of Patras Patras Greece

**Keywords:** biomarkers, brentuximab vedotin, chemotherapy‐induced peripheral neurotoxicity, neurofilament light chain, oxaliplatin, paclitaxel

## Abstract

**Background and purpose:**

A real‐time biomarker in chemotherapy‐induced peripheral neurotoxicity (CIPN) would be useful for clinical decision‐making during treatment. Neurofilament light chain (NfL) can be detected in blood in the case of neuroaxonal damage. The aim of the study was to compare the levels of plasma NfL (pNfL) according to the type of chemotherapeutic agent and the severity of CIPN.

**Methods:**

This single‐center prospective observational longitudinal study included patients treated with paclitaxel (TX; *n* = 34), brentuximab vedotin (BV; *n* = 29), or oxaliplatin (PT; *n* = 19). All patients were assessed using the Total Neuropathy Score–clinical version and Common Terminology Criteria for Adverse Events before, during, and up to 6–12 months after the end of treatment. Nerve conduction studies (NCS) were performed before and after chemotherapy discontinuation. Consecutive plasma samples were analyzed for NfL levels using a Simoa^®^ analyzer. Changes in pNfL were compared between groups and were eventually correlated with clinical and NCS data. Clinically relevant (CR) CIPN was considered to be grade ≥ 2.

**Results:**

Eighty‐two patients, mostly women (59.8%), were included. One third of the patients who received TX (29.4%), BV (31%), or PT (36.8%) developed CR‐CIPN, respectively, without differences among them (*p* = 0.854). Although pNfL significantly increased during treatment and decreased throughout the recovery period in all three groups, patients receiving TX showed significantly greater and earlier changes in pNfL levels compared to the other agents (*p* < 0.001).

**Conclusions:**

A variable change in pNfL is observed depending on the type of agent and mechanism of neurotoxicity with comparable CIPN severity, strongly implying the need to identify different cutoff values for each agent.

## INTRODUCTION

Chemotherapy‐induced peripheral neurotoxicity (CIPN) is the most common neurological complication in cancer patients. When cancer patients are treated with neurotoxic agents, there is a risk of misestimating patients' symptoms, which might lead to inappropriate treatment decisions and/or detrimental effects on the patients' quality of life. To date, there is no blood biomarker that can reliably indicate subclinical or predict severe neurotoxicity in this population. Light chain neurofilament (NfL), an axonal protein, has emerged as a promising marker of real‐time disease activity to monitor neurological diseases including injury of the peripheral nervous system by providing useful information of nerve tissue damage severity [1, 2, 3]. NfL has recently been investigated in cancer patients receiving neurotoxic agents, and previous research evidence demonstrates that NfL levels increase in close relation to the severity of CIPN during chemotherapy and decline after its cessation [4, 5, 6, 7, 8, 9, 10] (Table S1), clearly highlighting the potential role of blood NfL as a biomarker of CIPN evolution through time. Among chemotherapy drugs, antimicrotubule (anti‐MT) or platinum agents are among the most widely used neurotoxic agents and cause the vast majority of neuropathies seen in the clinical oncological practice. Platinum and anti‐MT agents cause peripheral neurotoxicity by damaging different targets resulting in an axonal neuropathy [6]. Oxaliplatin (PT) induces nuclear and mitochondrial DNA damage on dorsal root ganglia neurons, leading to neuronal dysfunction or death [11, 12]. MT agents including paclitaxel (TX), and vedotin‐based agents interfere with MT assembly, leading to a dying‐back axonopathy [13, 14]. In addition, a different degree and temporal profile of NfL level increase in rat models of CIPN have been reported in vivo [15].

Currently, it is unknown whether differences in the primary mechanism of neurotoxicity are associated with a variable degree of NfL release in humans. The aim of this study was to compare the change in plasma NfL (pNfL) over time in cancer patients receiving different types of neurotoxic chemotherapy. In addition, we also sought to explore the association between CIPN and pNfL after completion of chemotherapy to investigate its usefulness in the follow‐up of patients suffering from CIPN.

## MATERIALS AND METHODS

### Study design and recruitment

Adult patients with a definite diagnosis of breast cancer, gastrointestinal cancer, or lymphoma scheduled to receive TX‐based chemotherapy, PT, or brentuximab vedotin (BV), respectively, were prospectively recruited at the Hospital Universitari de Bellvitge–Institut Català d'Oncologia L'Hospitalet and were eventually enrolled in this single‐center longitudinal observational study. All chemotherapy schedules were administered according to standard doses and institution protocols. Briefly, all patients being treated with TX were women with breast cancer receiving up to 12 weekly cycles of TX. The FOLFOX regimen, having PT as the basic cytotoxic agent, was administered to colorectal (*n* = 17) and pancreatic (*n* = 2) cancer patients. BV‐based chemotherapy was administered as monotherapy (*n* = 11) or in combination with other chemotherapeutic agents (*n* = 17), depending on the type of lymphoma. Patients with Karnofsky performance score < 70, central nervous system metastases, or intake of any medication or presence of clinical disease that could interfere with clinical assessments were excluded. Uncomplicated diabetes or subclinical polyneuropathy at baseline was not considered an exclusion criterion. Patients were clinically evaluated at five subsequent time points: (i) before the initiation of chemotherapy (T0), (ii) at midtreatment (T1), (iii) after completion of chemotherapy (T2), and (iv) at 3 (T3) and (v) between 6 and 12 months (T6_12) after completion of treatment. The period between T0 and T2 was defined as CIPN development, whereas that between T2 and T6_12 is considered the CIPN recovery period. The ethics committee of the Hospital Universitari de Bellvitge–Institut Català d'Oncologia de L'Hospitalet approved the study (PR321/20). All participants gave written informed consent in accordance with the Declaration of Helsinki before entering the study.

### Neurological assessment

The chronic CIPN form was defined as a painful or painless clinical syndrome characterized by evidence of dose‐related, symmetrical sensory abnormalities lasting at least two subsequent chemotherapy cycles without a “symptoms free” interval [13]. Patients were tested for CIPN incidence and severity by means of the seven‐item composite Total Neuropathy Score–clinical version (TNSc; John Hopkins University). The TNSc is a validated composite measure that includes the patient's symptom report and a neurological assessment including examination of vibration and pinprick sensation, deep tendon reflexes, and manual muscle testing; a higher score indicates more severe neuropathy [16]. The following grading cutoffs were used: grade I (score = 1–7), grade II (score = 8–14), grade III (score = 15–21), and grade IV (score > 21) [17].

At each visit, the presence and severity of motor and sensory neurotoxicity was collected according to the Common Terminology Criteria for Adverse Effects version 5 [18]. In summary, CIPNs are classified as grade 1, reflecting mild symptoms that do not require specific intervention; grade 2, indicating moderate events affecting instrumental activities of daily living (ADL); or grade 3, indicating severe symptoms affecting self‐care and ADL. Conventional nerve conduction studies (NCS) were performed before chemotherapy (T0) and ~4 weeks after the last cycle (T2), using a Nicolet EDX Synergy device (Natus Medical, Pleasanton, CA, USA). NCS were performed bilaterally on the sural and radial sensory nerves, and right peroneal and median motor nerves, employing standard methods to perform surface stimulation and recording [19, 20]. The widely accepted criteria for identifying abnormalities were employed as previously described [5, 21]. The diagnosis of CIPN at T2 was based on symptoms (complaints of sensory symptoms in fingertips or glove and stocking distribution compatible with neuropathy lasting for >1 week), neurological signs, and NCS according to established consensus criteria consistent with a distal symmetric polyneuropathy [22].

### NfL concentration

Venous blood was collected in the morning and centrifuged at 3000 rpm for 10 min. Plasma was stored at −80°C and thawed immediately before use. Samples were analyzed retrospectively. NfL levels were analyzed longitudinally in duplicate in plasma in a blinded fashion using the Simoa HD‐1 ultrasensitive single molecule analyzer (Quanterix, Lexington, Boston, MA, USA). Samples were examined following the manufacturer's instructions with appropriate internal standards and controls. Baseline pNfL levels were compared with age‐related clinical benchmark cutoff points [23, 24, 25]. In the subgroup of patients receiving TX, with the aim to investigate differences between plasma and serum NfL, we performed a comparative analysis of paired baseline present pNfL and previously reported serum NfL [4]. Although clinical and neurophysiological data from some TX patients have previously been reported [4, 5], data on pNfL and long‐term CIPN in this population are novel and have never been reported.

### Statistics

The study data were collected and stored using RedCAP, hosted at Bellvitge Biomedical Research Institute. Descriptive statistics presented categorical variables as observed counts and weighted percentages, and continuous variables as mean or median with the corresponding standard error or range, according to their nature. Patients were classified according to the severity of CIPN as not clinically relevant (termed “NCR‐CIPN”; National Cancer Institute Common Toxicity Criteria [NCI‐CTC] grade < 2) or clinically relevant (CR‐CIPN; NCI‐CTC ≥ 2) at the end of chemotherapy treatment. As mentioned earlier, two distinct periods were considered separately for the analysis: CIPN development (T0–T2) and recovery period (T2–T6_12). For categorical dichotomic variables, we used a χ^2^ test. To quantify the differences between patient groups at baseline, one‐way analysis of variance (ANOVA) with post hoc analysis and Kruskal–Wallis tests was used, depending on the nature of the variable and after checking for normality using the Kolmogorov–Smirnov test. The repeated measures ANOVA test was used to evaluate differences between groups and time. The association between clinical and blood parameters was assessed using Spearman correlation coefficient. Statistical analyses were performed with SPSS Statistics version 25.0 (IBM, Armonk, NY, USA) and Prism version 9.4.1 (GraphPad Software, San Diego, CA, USA). Graphs were created with Prism. Statistical significance was set at *p* < 0.05.

## RESULTS

Of the 91 patients who were prospectively recruited, overall, 85 were eventually analyzed. Six patients were excluded because of unavailability of their pNfL samples at baseline (before treatment). In addition, three patients with abnormal pNfL values, (compared to age‐ and gender‐related clinical benchmark cutoff points) at baseline due to recent neurological events were excluded. Finally, 82 patients were included (Figure S1). Demographic, clinical, neurophysiological, and pNfL data in regard to chemotherapy agent are summarized in Table 1. None of the participants reported symptoms of neuropathy prior to chemotherapy treatment. However, subclinical (grade 1) peripheral neuropathy was identified in seven (8.75%) patients prior to chemotherapy, mostly in the oxaliplatin‐treated patients (TX, *n* = 2; BV, *n* = 1; and PT, *n* = 4), all of them having pNfL within normal ranges. Significant differences in baseline NCS recordings were observed among patients according to chemotherapy groups, consistent with the expected differences in NCS due to age‐related variability (Table 1). No differences in baseline pNfL among chemotherapy groups were observed. The pNfL at baseline correlated with age (*r* = 0.526, *p* < 0.001) but not thereafter (at T1, T2, T3) until T6 (T6: *r* = 0.359, *p* = 0.040). No correlation with body mass index (BMI; *r* = −0.126, *p* = 0.4) was identified. In the TX group, serum and pNfL were very highly correlated (*r* = 0.823, *p* < 0.001), being slightly higher (∼10%) in serum (17.07 ± 11.83 pg/mL) than EDTA plasma (15.37 ± 13.94 pg/mL).

**TABLE 1:** Demographic, clinical, serological, and neurophysiological characteristics before chemotherapy (T0).

Characteristic	MT‐disrupting agents		Platinum agent	*p*
	Brentuximab vedotin	Paclitaxel	Oxaliplatin	
	*n* = 29 (35.4%)	*n* = 34 (41.5%)	*n* = 19 (23.2%)	
Age, years [range]	49 [21–93]	50 [31–69]	61 [43–76]	0.021
Gender, men/women	19/10	0/34	14/5	<0.0001
Body mass index	27.4 ± 5.4	26.25 ± 4.83	27.8 ± 5.3	0.537
TNSc	0 [0–5]	0 [0–4]	0 [0–7]	0.014
NCI‐CTC	0 [0–1]	0 [0–1]	0 [0–1]	0.086
Plasma NfL, pg/mL	15.03 ± 12.32	16.20 ± 13.62	13.64 ± 6.35	0.748
Sural SNAP right, μV	15.19 ± 6.26	16.05 ± 7.46	10.57 ± 4,89	0.033
Sural SNAP left, μV	15.63 ± 6.58	16.55 ± 6.99	11.83 ± 5.43	0.077
Radial SNAP right, μV	28.91 ± 11.78	33.24 ± 12.95	21.89 ± 7.96	0.003
Radial SNAP left, μV	34.05 ± 21.87	32.35 ± 13.83	21.63 ± 7.49	0.024
Median CMAP, mV	10.27 ± 3.32	11.01 ± 3.25	9.36 ± 1.53	0.337
Peroneal CMAP, mV	5.92 ± 2.76	4.89 ± 2.09	6.14 ± 2.26	0.200

Abbreviations: CMAP, compound muscle action potential; MT, microtubule; NCI‐CTC, National Cancer Institute Common Toxicity Criteria; NfL, light chain neurofilament; SNAP, sensory nerve action potential; TNSc, Total Neuropathy Score–clinical version.

**FIGURE 1:**
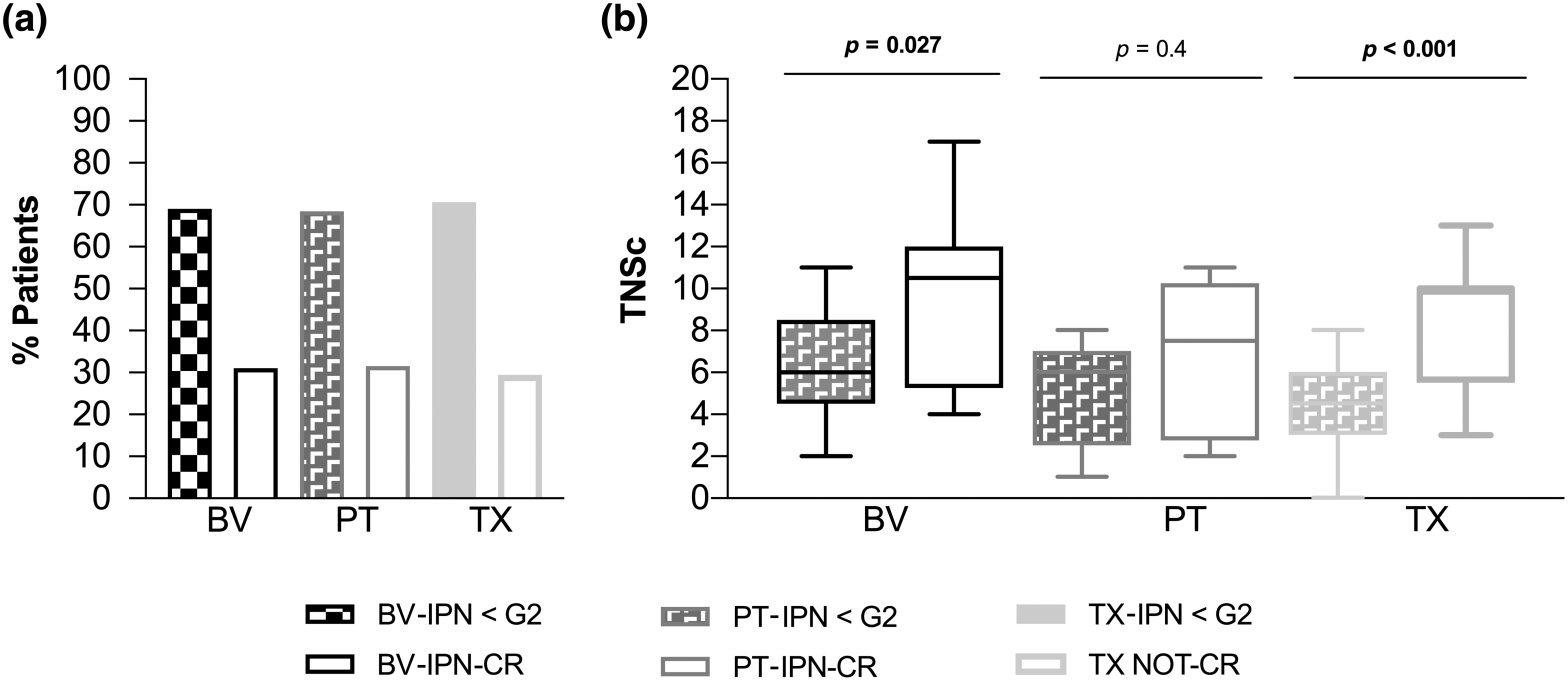
(a). Rate of having chemotherapy‐induced peripheral neurotoxicity (CIPN; clinically relevant [CR]) or not (<grade 2 [G2]), according type of agent (brentuximab [BV]: black; oxaliplatin [PT]: grey; paclitaxel [TX]: light grey) at the end of treatment (T2). (b) Total Neuropathy Score per each agent on finishing treatment (T2) and according to severity of CIPN (CR or not). Results are expressed as median (range). IPN, induced peripheral neurotoxicity; TNSc, Total Neuropathy Score–clinical version.

**FIGURE 2:**
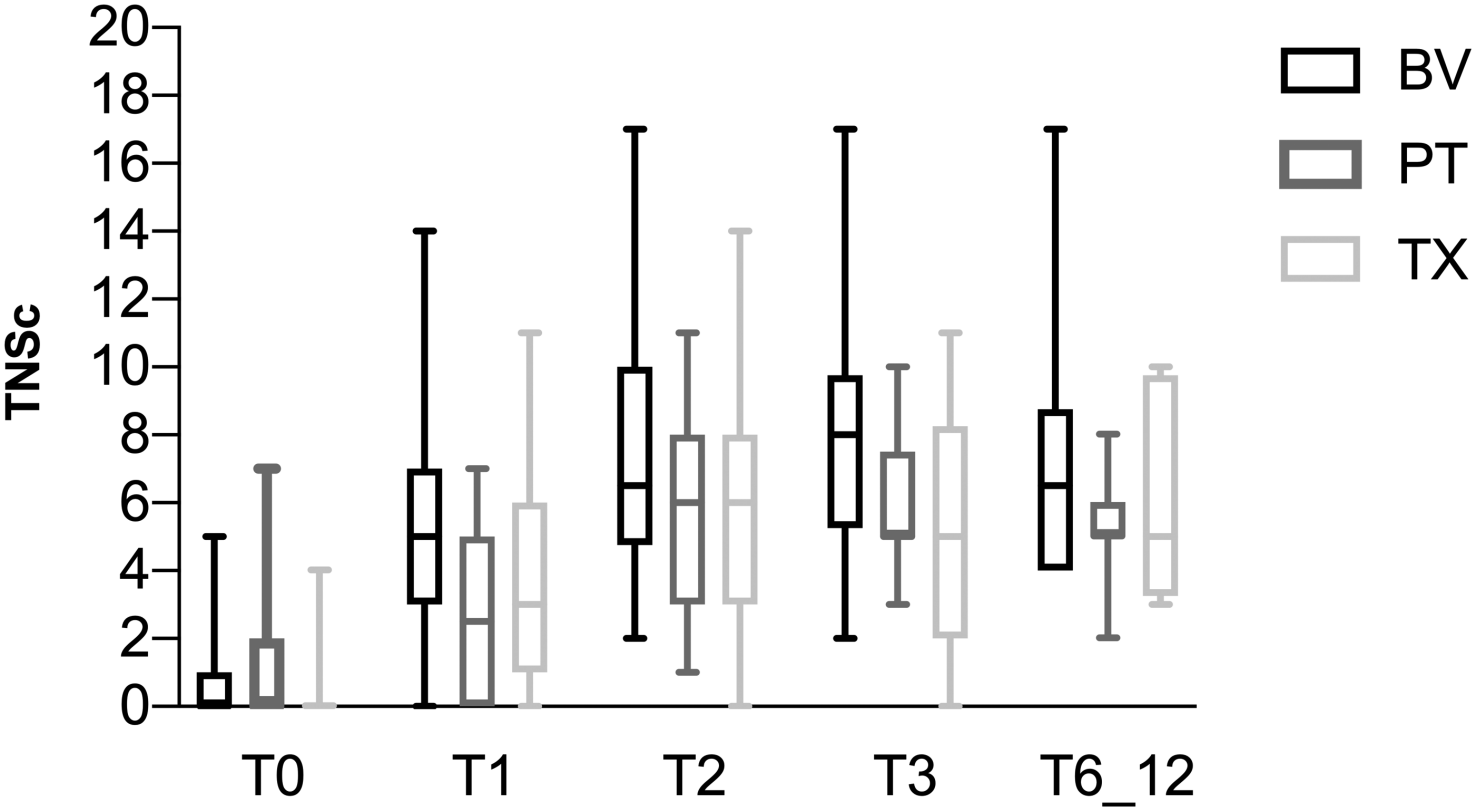
Total Neuropathy Score–clinical version (TNSc) per each agent over the chemotherapy‐induced peripheral neurotoxicity development (T0–T2) and recovery (T2–T6_12) periods (brentuximab [BV]: black; oxaliplatin [PT]: grey; paclitaxel [TX]: light grey). Results are expressed in median (range).

One third of the whole series developed CR‐CIPN (*n* = 26, 31.7%), with no differences in the rate of CR‐CIPN according to the type of agent, TX (29.4%), BV (31%), or PT (36.8%; *p* = 0.854; Figure 1a) or by the mechanism of neurotoxicity (MT [19/61, 31.14%] or PT [7/19, 36.8%], *p* = 0.585). In the whole series, TNSc scores significantly increased during the treatment period (*p* < 0.001), whereas no significant changes were identified over the recovery period (*p* = 0.439; Figure 2). In the postchemotherapy period, patients displaying CR‐CIPN due to MT‐targeted agents had higher TNSc scores, compared to NCR‐CIPN (Figure 1b). Among those with CR‐CIPN, no significant differences in TNSc at T2 were identified, according to the agent type (*p* = 0.199).

**FIGURE 3:**
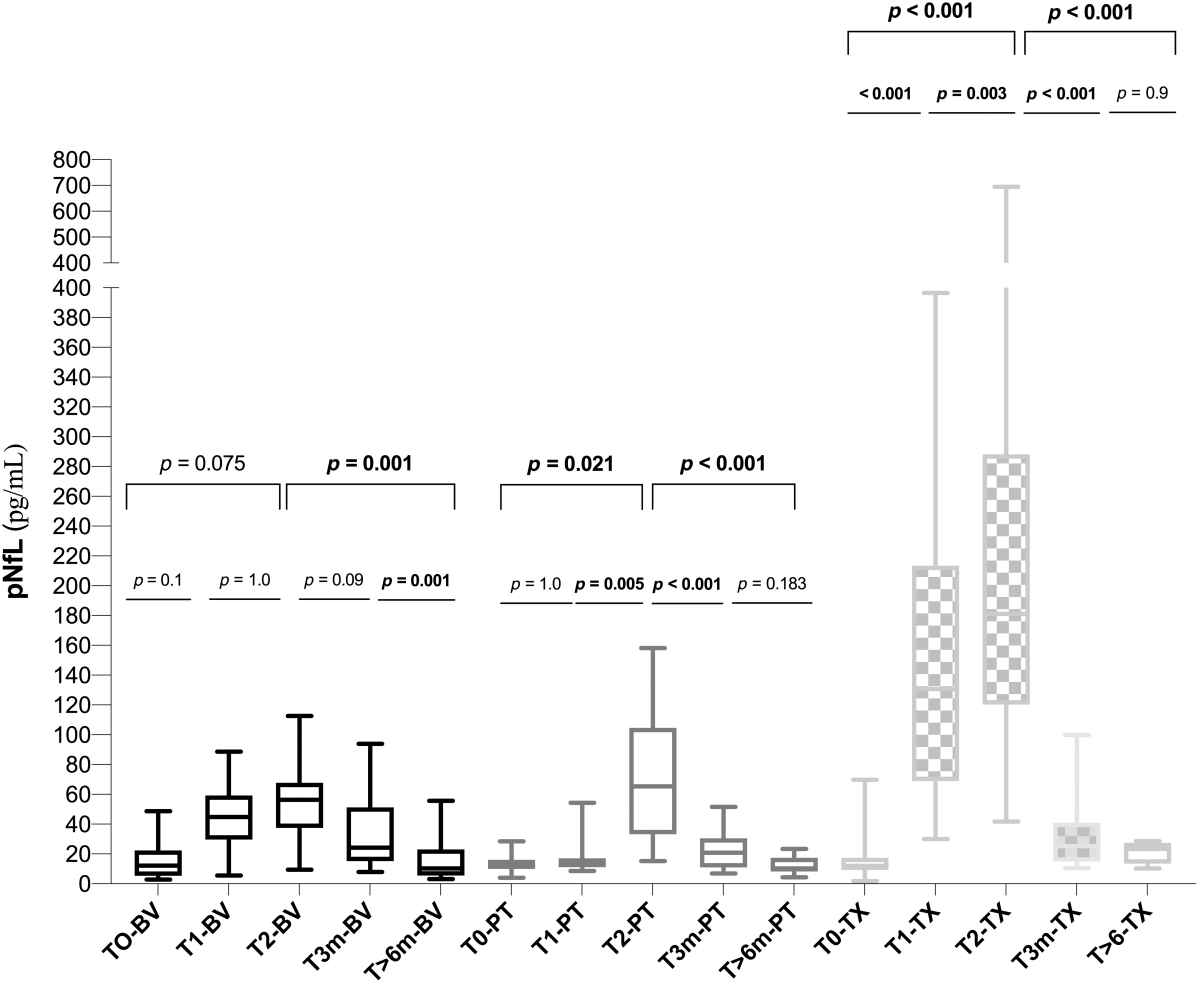
Plasma neurofilament light chain (pNfL) levels over the course of treatment and follow‐up period. Data are mean and range. Probability values were calculated by repeated measures analysis of variance. BV, brentuximab; PT, oxaliplatin; TX, paclitaxel; T0, Baseline; T1: Mid‐treatment; T2: After last cycle; T3m: 3 months after last cycle; T>6m: 6 or more months after last cycle.

**FIGURE 4:**
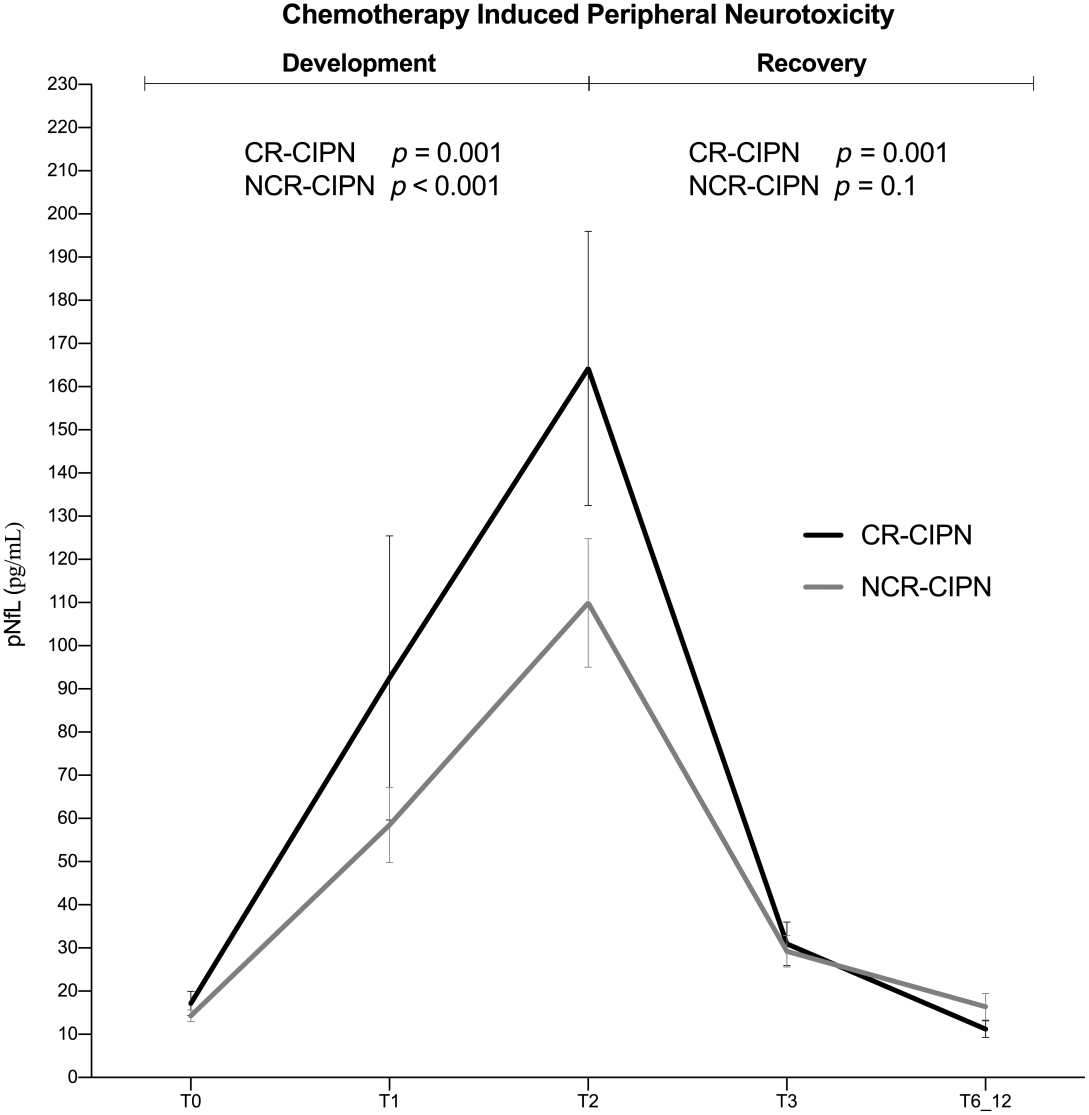
plasma neurofilament light chain (NfL) (pNfL) according severity of chemotherapy‐induced peripheral neurotoxicity (CIPN) on finishing chemotherapy (pNfL levels correspond to mean values and SEM). CR, clinically relevant; NCR, not clinically relevant.

In each group of patients, pNfL levels increased over treatment, and rapidly decreased between chemotherapy treatment and the first 3‐month follow‐up, with a milder delayed decline (Figure 3). An early increase at midtreatment was only observed in patients receiving TX, compared to BV or PT (*p* < 0.001; Figure 3). At chemotherapy completion, the increase in pNfL values remained significant in the TX group compared to the other two groups (*p* < 0.001). Likewise, the levels of pNfL in the TX group at T1 and T2 were higher compared to BV (*p* < 0.001) or PT (*p* < 0.001). No differences in the amount of pNfL between BV and PT were identified at T1 (*p* = 0.401) and T2 (*p* = 0.1), respectively. The differences in NfL levels at finishing chemotherapy (T2) between patients treated with BV (alone vs. in combination) were not significant (49.1 vs. 65.9; *p* = 0.095). Nonetheless, a significant change in pNfL was observed during chemotherapy and at the recovery period in both the CR‐CIPN and NCR‐CIPN groups (Figure 4), without evidence of differences in the pNfL levels between the severity groups at any time of assessment.

During the CIPN development period, between T0 and T2, the TNSc scores were correlated with pNfL in the TX group (*r* = 0.551, *p* < 0.001) and in the BV group (*r* = 0.556, *p* < 0.001), having a lower albeit significant correlation in the PT group (*r* = 0.341, *p* = 0.01). No correlation between pNfL and TNSc was identified for any agent during the recovery period (data not shown).

**FIGURE 5:**
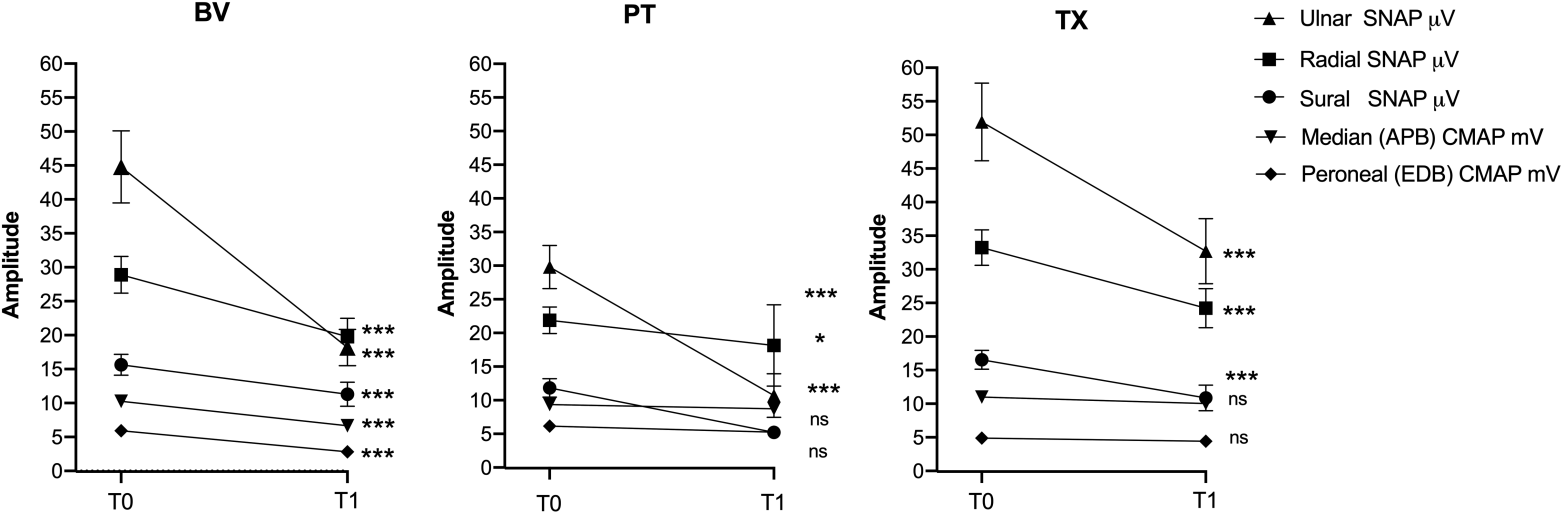
Change in the mean neurophysiological results obtained before (T0) and at the end of treatment (T1) in (from up to down): ulnar, radial, and sural sensory nerve action potential (SNAP) amplitude, and median and peroneal compound muscle action potential (CMAP) registered in abductor pollicis brevis (APB) and extensor digitorum brevis (EDB), respectively. *p < 0.05, ***p < 0.001. BV, brentuximab; ns, not significant; PT, oxaliplatin; TX, paclitaxel.

In the whole series, the percentage of abnormalities in the amplitudes of sensory nerve action potentials (SNAPs) in lower limbs after chemotherapy was higher in patients with CR‐CIPN (right sural SNAP: 58.5% vs. 37.9%, *p* = 0.028; left sural SNAP: 61.1% vs. 36.8%, *p* = 0.028) and peroneal motor compound muscle action potential (CMAP; 53.8% vs. 30.5%, *p* = 0.025). After chemotherapy, a significant decrease in SNAPs was identified in the three chemotherapy groups. Patients receiving BV had a significant decrease in CMAPs of median and peroneal nerves. A trend (*p* = 0.059) in the change of lower leg CMAP was observed in those patients receiving TX. No differences in the amplitude of motor nerves was observed in patients receiving PT (Figure 5). There were no significant differences in NCS abnormalities according to the type of chemotherapy (TX vs. BV vs. PT) and mechanism to evoke CIPN (MT vs. PT).

## DISCUSSION

We clinically demonstrate that pNfL dynamically varies depending on the type of offending agent, the mechanism by which cytotoxic agents evoke neurotoxicity, and timing of assessment. We also show that the pNfL increments correlate with the clinical severity of CIPN during chemotherapy. Overall, pNfL was preferentially increased in subjects who reported moderate/severe CIPN, compared to subjects with minimal CIPN. However, among those patients displaying CR‐CIPN at the end of chemotherapy, those who were TX‐treated had a significantly higher increase in their pNfL in comparison with patients treated with the other studied type of anti‐MT agent (i.e., BV) or a PT‐based agent, such as PT. Besides, the timing of pNfL changes over the treatment period also differed according to the drug type. In the first period, when patients still did not have overt CIPN symptoms and/or signs, we observed an earlier pNfL increment in patients receiving MT‐based agents, and particularly in patients treated with TX compared to the other two cytotoxic agents. These results are in agreement with previous findings reported by our group demonstrating that NfL quantification before midtreatment (after 2–3 chemotherapy courses) might be able to early predict the final outcome of taxane‐induced peripheral neurotoxicity in patients without or with evidence of mild peripheral nerve damage [4].

Our findings are consistent with previous preclinical evidence in rat models, where higher levels were disclosed in TX‐treated compared with PT‐treated rats [15]. Differences in the course of NfL level changes have also been reported in another experimental study, which showed that the corresponding NfL levels in TX‐treated rats picked up earlier compared to PT‐treated animals [15].

On clinical grounds, our results are in keeping with findings of two previous studies including patients treated with TX [10] or PT [9]. In the study where TX‐treated patients developed severe CIPN, the serum NfL levels (mean = 506 pg/mL) [10] were higher than in patients treated with PT (mean = 373.4 pg/mL) [9]. Nonetheless, the concurrent treatment with another platinum‐based agent (carboplatin) in patients receiving TX could likely have augmented the development of CIPN, and consequently this confounding factor might bias the results in keeping with the increase in sNfL observed in Kim et al. study and most of the previously reported studies (Table S1) including patients treated with a combination of chemotherapy agents (MT and PT) [8, 10, 26, 27]. In addition, the delayed increase of pNfL in patients receiving PT has also been previously observed. The sNfL in a cohort of 34 cancer patients who were treated with PT‐based regimen showed a mild increase in sNfL concentrations between baseline and 3 months of treatment (median = 22.3 pg/mL), but more prominent changes were observed between 3 and 6 months during chemotherapy (median = 115.0 pg/mL) [9]. These findings bolster the potential role of NfL as a promising early biomarker of subclinical CIPN in patients treated with MT‐targeted agents, particularly TX. The same applies, apparently to a lesser degree, for patients treated with PT‐based chemotherapy drugs.

The severity of CIPN typically declines postchemotherapy, but it can be persistent in severely affected patients, taking long to resolve following the cessation of treatment or even remaining indefinitely [28]. The current study showed that increases in pNfL were associated with worst CIPN severities, according to TNSc grading during chemotherapy, but not in the postchemotherapy period, which is consistent with previous evidence showing that elevated levels of sNfL return to normal several months after stroke or traumatic brain injury, but remain relatively stable throughout the course of neurodegenerative diseases [29]. Our data show that pNfL levels decreased early after finishing chemotherapy and were slightly changed throughout the follow‐up study period. Particularly, within the “coasting period,” when symptoms may progress for several weeks after the cessation of the drug, there were no increases in pNfL levels. Hence, even if CIPN persists, this is no longer reflected by pNfL levels, which mostly reached normal ranges at the last follow‐up we performed. The current prospective study, having one of the longest time frames among CIPN follow‐up studies, supports that the increased pNfL levels indicate ongoing axonal damage under the noxious toxic stimulus, and as such we advocate the view that pNfL clinically warrants being assessed during chemotherapy, whereas measuring pNfL levels postchemotherapy lacks clinical relevance, as it is of insignificant importance in monitoring the recovery of CIPN.

In the present study, the NCS findings correlated with findings consistent with clinically relevant CIPN. However, although experimental findings identified that NfL measurements discriminated between a length‐dependent axonopathy and primary neuronopathy induced by TX and cisplatin in rats, respectively, we were unable to identify such differences in the degree of axonal loss among the different chemotherapy regimens or according to the pathogenetic mechanism of neurotoxicity. Besides the moderate sample size of our enrolled patients, other reasons may account for this lack of differences, mainly including that NCS can detect large fiber damage but are insensitive to changes in small diameter nerve fibers, which might also contribute to pNfL release [30], although conflicting results are emerging in the literature [31].

The NfL assay is gradually becoming an essential diagnostic tool for the diagnosis of many neurological diseases or conditions and is increasingly introduced into clinical laboratory routine. However, the application of NfL measurement as a tool for clinical decision‐making and research in cancer patients undergoing neurotoxic chemotherapy still has limitations due to gaps in knowledge concerning the factors that might bias the NfL measurements. In our study, as expected, age was positively correlated with NfL levels [32]. However, we confirm that the cancer per se does not evoke neuroaxonal degeneration. Furthermore, although several studies have reported an inverse correlation between BMI and NfL levels [33], we have not identified such an association between pNfL and BMI prechemotherapy. Recently, NfL expression has been reported to be altered in sarcopenic patients [34]. Although BMI can provide some indication of overall body composition, it does not directly measure muscle mass or strength, which are critical factors in determining the risk of sarcopenia. It remains obscure whether cancer‐associated sarcopenia can underlie our negative results, and as such this assumption requires further study.

A predictive biomarker of dose‐limiting CIPN could be used to personalize the chemotherapy dosing regimen to improve both safety and efficacy of chemotherapy. To date, neurological monitoring with clinical [35] and neurophysiological [21] biomarkers was deemed useful. However, neurophysiological, nerve imaging, and other techniques to assess CIPN are time‐consuming and often not feasible for routine oncologic care [36]. Because blood measurements of biomarkers are minimally invasive and can thus be repeated over regular periods of time, they are particularly suitable for longitudinally monitoring CIPN in cancer patients in whom routine frequent blood tests are performed anyhow as standard of care to assess hematological and other organ toxicities. The present study, in which the plasma sample was chosen for technical (availability) rather than scientific reasons, demonstrates the feasibility of pNfL in this context.

Our study has some limitations and strengths. First, the small sample size of each group, including different types of lymphoma in the BV group, and lack of a control healthy group should be acknowledged as limitations. Besides, the criteria for CIPN classification were based on the NCI‐CTC. Despite its known limitations [37], which represent the underlying motivation for the present study in identifying more objective biomarkers, a physician‐rated parameter like the NCI‐CTC represents the universally used and currently standard means of CIPN gradation in oncologic clinical practice. Furthermore, the cutoff at grade ≥ 2 severity of CIPN was considered due to its critical value in guiding clinical decision‐making. The lack of a patient‐reported outcome measure might also be a limitation of our study in clinical translation.

These limitations aside, we applied a longitudinal comparative analysis including detailed clinical and, in most of the patients, neurophysiological assessment. Very few longitudinal studies have been reported to date (Table S1) to apply methodology similar to ours. The timing of assessment in scheduled intervals rather than similar timings of assessment should also be highlighted as a strength from an oncologic perspective. However, NfL change for various therapeutic regimens may differ from the single regimen examined in the present study. Furthermore, two of the drugs evaluated in the present study (PT and TX) are first‐line treatments in the most frequently diagnosed neoplasms worldwide and cause the vast majority of neurotoxicity events in the clinical oncological practice, which implies the vast clinical applicability of the results we herein present. Additionally, the comparison with novel agents, such as BV, allows us to also report on agents with a priori shared mechanism of action. Our study contributes to our understanding of the pathological changes underlying CIPN, suggesting that additional mechanisms beyond the axonal damage may be associated with BV‐induced neuropathy.

To conclude, pNfL is an easily accessible and objective biomarker of CIPN that can variably change, according to the type of the neurotoxic agent and during chemotherapy, in close association with the clinical severity of CIPN. Therefore, before implementing pNfL as a biomarker of ongoing CIPN for diagnostic and monitoring purposes, further studies are warranted to better define cutoff values for each agent or even chemotherapy schedule. In contrast, the measurement of pNfL appears not to be useful postchemotherapy to monitor the CIPN recovery.

## AUTHOR CONTRIBUTIONS

**Roser Velasco:** Conceptualization; methodology; data curation; investigation; validation; formal analysis; supervision; funding acquisition; writing – original draft; writing – review and editing; resources; project administration; visualization. **Andreas A. Argyriou:** Writing – review and editing; writing – original draft; supervision. **German Ferrer:** Writing – review and editing; data curation; investigation. **Jordi Bruna:** Writing – original draft; funding acquisition; methodology; writing – review and editing. **Eva Domingo‐Domenech:** Writing – review and editing; investigation. **Carla Marco:** Investigation; writing – review and editing; data curation; visualization. **Agostina Stradella:** Writing – review and editing. **Berta Laquente:** Writing – review and editing; investigation. **Cristina Santos:** Writing – review and editing; investigation.

## CONFLICT OF INTEREST STATEMENT

Roser Velasco consultancy and speaker for Takeda. Eva Domingo‐Domenech consultancy for Takeda; speaker bureau for Takeda; travel grants from Takeda. Other authors do not report any conflict of interest to disclose.

## Supporting information


Figure S1.

Table S1.

## Data Availability

The datasets generated and/or analyzed during the current study are available from the corresponding author on reasonable request from any qualified investigator.
