# Supplementary figures and images for: Plasma neurofilament light chain levels in chemotherapy‐induced peripheral neurotoxicity according to type of anticancer drug
Source: Eur J Neurol. 2024 Jul 1;31(9):e16369. doi: 10.1111/ene.16369 (PMC11295167; doi:10.1111/ene.16369)

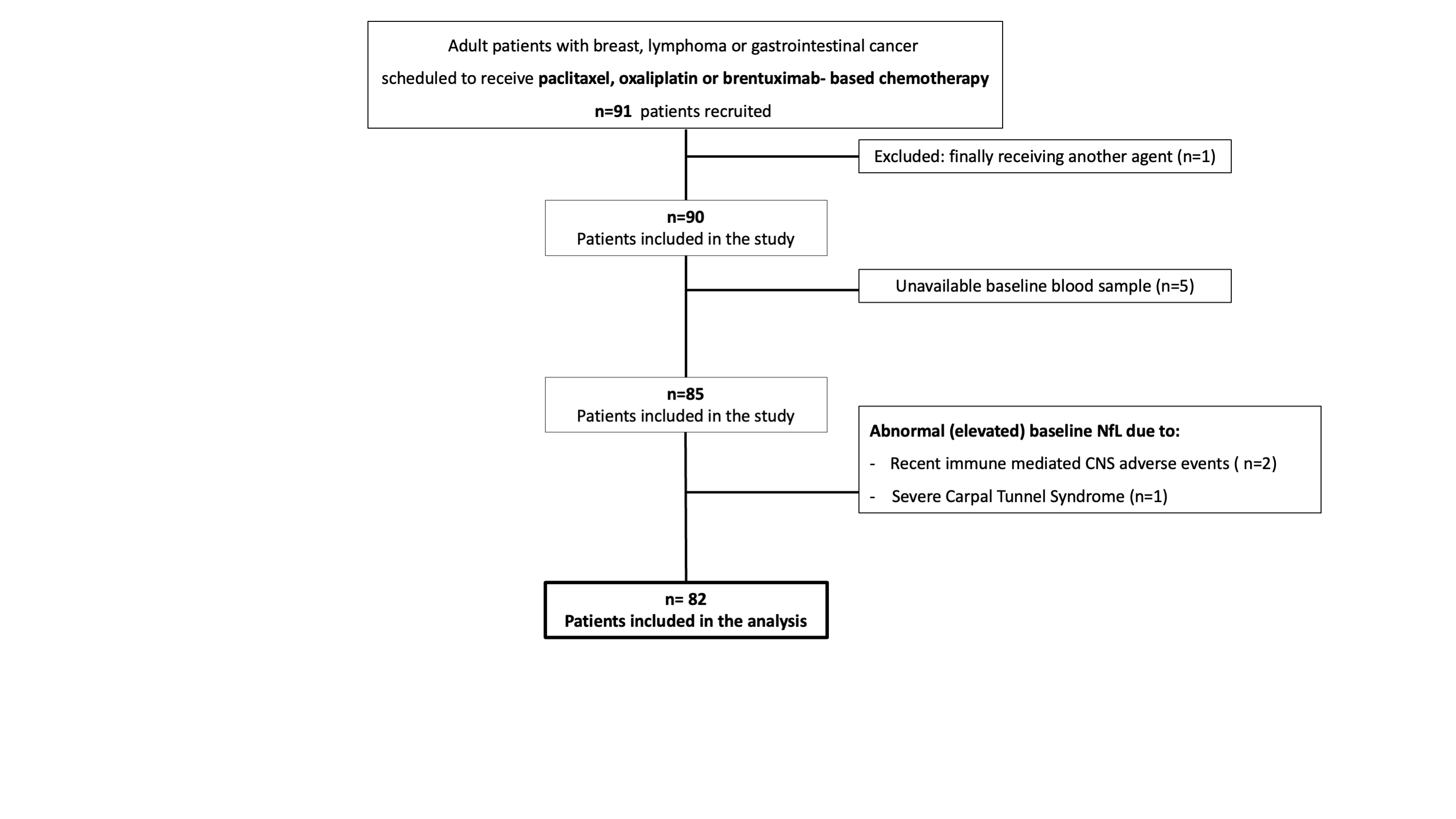

Supplement: Supplementary file 1 — Figure S1. [file ENE-31-e16369-s001.tiff]
