# Supplementary material for: Plasma neurofilament light chain levels in chemotherapy‐induced peripheral neurotoxicity according to type of anticancer drug
Source: Eur J Neurol. 2024 Jul 1;31(9):e16369. doi: 10.1111/ene.16369 (PMC11295167; doi:10.1111/ene.16369)
Supplement: Supplementary file 2 — Table S1. [file ENE-31-e16369-s002.docx]

**Table Supplementary 1**. Studies evaluating Neurofilament Light Chain in patients with chemotherapy induced peripheral neurotoxicity.

| **Author, Year** | **Chemo Drug** | **Type of Study** | **N (patients)** | **Timing of NfL samples** | **Neurological assessment** | **Main results** |
| --- | --- | --- | --- | --- | --- | --- |
| **Cebulla,** 2023 | Bortezomib | Cross-sectional | 70  (10 controls) | 1 | Sensory and motor  function, QST, NCS  CTC-NCI v4 | * sNfL levels were higher in patients compared to controls  *A trend towards an increase in sNfL across neuropathy severity  grades in patients under treatment  *sNfL correlated with sural SNAP in patients under BTZ treatment |
| **Burgess**, 2022 | Paclitaxel  + Carboplatin | Retrospective analysis of a clinical trial | 88 | 2  At baseline and 21 days after the first of six cycles of chemo | CTC-NCI v4 | sNfL changes early in treatment can predict dose-limiting CIPN prior to onset of symptoms |
| **Benashley,**  2022  (Abstract) | Paclitaxel | Prospective | 21 | Every two weeks | CTCAE  FACTGOG-Ntx | sNfL and CIPN symptoms increased concurrently during treatment |
| **Mortensen,** 2022 | Paclitaxel  + carboplatin | Retrospective study cohort | 190 | Baseline and after each of the following 2 or 6 cycles | CTCAE | Patients with sNFL>150 pg/mL after  first cycle had increased risk to discontinue paclitaxel early |
| **Huehnchen,** 2022 | Paclitaxel  + /-  carboplatin | Prospective | 17 (PTX)  14 (PTX+CPL)  25 (no chemotherapy)  6 (healthy controls) | 2  before  chemotherapy (V1) and after 28 weeks (V2, after chemotherapy) | Total Neuropathy Score reduced (TNSr)  (TNS + NCS)  EORTC-QLQ-CIPN20  CTCAE | sNFLs strongly increased in  patients with CIPN, but not in patients receiving chemotherapy without CIPN or  controls. |
| **Karteri,** 2022 | Paclitaxel | Prospective | 65 | 4  (baseline), after  2 (week 2) and 3 (week 3) weekly courses, and at the end of chemotherapy (week  12). | TNSc | sNfL levels were increased along treatment and were significantly higher both during and after completion of treatment, in patients with grade 2-3 PIPN, compared to those with grade 0-1, while the mean levels of sNfL were significantly correlated  with clinical severity of PIPN.  sNfL, (85 pg/mL in particular as a cutoff value after delivering 3 weekly paclitaxel courses), in early and independent prediction of development of grade 2-3 PIPN after treatment  cessation. |
| **Velasco,**  2022 | Paclitaxel | Prospective | 27 | 2  Before and after | TNS  NCS | sNfL levels increase proportionally  to the degree of sensory NCS abnormalities |
| **Kim,** 2022 | Paclitaxel  + Carboplatin | Prospective | 48 | 6 | NCI-CTC  EORTC QLQ-CIPN20  NCS | sNfL levels increased during paclitaxel treatment in all  patients.  After two, four, and six cycles, patients with grade 3 PIPN exhibited  higher mean sNfL levels than those in the 0–2 grade range (p = 0.004, p = 001, and p < 0.001, respectively). |
| **Kim,**  2020 | Oxaliplatin | Prospective | 34 | 3  baseline, 3 months, and 6 months of treatment | NCI-CTC  NCS  EORTC-QLQ-CIPN20 | Patients with grade-3 OIPN showed  significantly higher mean sNfL levels than patients with grade 0–2 OIPN. Obvious neuronal damage occurs only after 3 months of  oxaliplatin treatment. |
| NCI-CTC: National Cancer Institute-Common Toxicity Criteria; | | | | | | |
